# Versatile roles of protein flavinylation in bacterial extracyotosolic electron transfer

**DOI:** 10.1128/msystems.00375-24

**Published:** 2024-07-23

**Authors:** Shuo Huang, Raphaël Méheust, Blanca Barquera, Samuel H. Light

**Affiliations:** 1 Duchossois Family Institute, University of Chicago, Chicago, Illinois, USA; 2 Department of Microbiology, University of Chicago, Chicago, Illinois, USA; 3 Génomique Métabolique, CEA, Genoscope, Institut François Jacob, Université d'Évry, Université Paris-Saclay, CNRS, Evry, France; 4 Department of Biological Sciences, Rensselaer Polytechnic Institute, Troy, New York, USA; 5 Department of Chemistry and Chemical Biology, Rensselaer Polytechnic Institute, Troy, New York, USA; 6 Center for Biotechnology and Interdisciplinary Studies, Rensselaer Polytechnic Institute, Troy, New York, USA; Wageningen University, Wageningen, the Netherlands

**Keywords:** redox, electron transfer, flavin

## Abstract

Bacteria perform diverse redox chemistries in the periplasm, cell wall, and extracellular space. Electron transfer for these extracytosolic activities is frequently mediated by proteins with covalently bound flavins, which are attached through post-translational flavinylation by the enzyme ApbE. Despite the significance of protein flavinylation to bacterial physiology, the basis and function of this modification remain unresolved. Here we apply genomic context analyses, computational structural biology, and biochemical studies to address the role of ApbE flavinylation throughout bacterial life. We identify ApbE flavinylation sites within structurally diverse protein domains and show that multi-flavinylated proteins, which may mediate longer distance electron transfer via multiple flavinylation sites, exhibit substantial structural heterogeneity. We identify two novel classes of flavinylation substrates that are related to characterized proteins with non-covalently bound flavins, providing evidence that protein flavinylation can evolve from a non-covalent flavoprotein precursor. We further find a group of structurally related flavinylation-associated cytochromes, including those with the domain of unknown function DUF4405, that presumably mediate electron transfer in the cytoplasmic membrane. DUF4405 homologs are widespread in bacteria and related to ferrosome iron storage organelle proteins that may facilitate iron redox cycling within ferrosomes. These studies reveal a complex basis for flavinylated electron transfer and highlight the discovery power of coupling comparative genomic analyses with high-quality structural models.

**IMPORTANCE:**

This study explores the mechanisms bacteria use to transfer electrons outside the cytosol, a fundamental process involved in energy metabolism and environmental interactions. Central to this process is a phenomenon known as flavinylation, where a flavin molecule—a compound related to vitamin B2—is covalently attached to proteins, to enable electron transfer. We employed advanced genomic analysis and computational modeling to explore how this modification occurs across different bacterial species. Our findings uncover new types of proteins that undergo this modification and highlight the diversity and complexity of bacterial electron transfer mechanisms. This research broadens our understanding of bacterial physiology and informs potential biotechnological applications that rely on microbial electron transfer, including bioenergy production and bioremediation.

## INTRODUCTION

Essential aspects of prokaryotic physiology take place beyond the bounds of the cell cytosol. Extracytosolic interactions can occur at the outer leaflet of the plasma membrane, periplasm, cell wall, or surrounding environment. Within the extracytosolic environment, redox reactions (defined by the reduction of an electron acceptor and oxidation of an electron donor) represent an important class of activities that have functions in respiration, maintenance/repair of extracytosolic proteins, and assimilation of minerals (1–3).

Flavins are a group of small molecules that contain a conserved redox-active isoalloxazine ring system. Most microbes synthesize riboflavin (or vitamin B2), flavin mononucleotide (FMN), and flavin adenine dinucleotide (FAD). FMN and FAD serve as common co-factors within diverse redox-active enzymes (4). Flavinylation describes the covalent attachment of a flavin moiety to a protein and frequently occurs in proteins involved in extracytosolic electron transfer (5). The alternative pyrimidine biosynthesis protein, ApbE, is a widespread FMN transferase that flavinylates a conserved [S/T]GA[**S/T**]-like sequence motif (flavinylated amino acid in bold) within substrate proteins (6).

Extracytosolic proteins flavinylated by ApbE are integral for a number of extracytosolic electron transfer systems involved in bacterial bioenergetics. These include the cation-pumping NADH:quinone oxidoreductase (Nqr) and *Rhodobacter* nitrogen fixation (Rnf) complexes, nitrous oxide and organohalide respiratory complexes, and a Gram-positive extracellular electron transfer system (7–11). ApbE flavinylation has also been shown to mediate electron transfer to a group of related flavin reductases that use a variety of metabolites as respiratory electron acceptors (12–15).

Using the presence of ApbE and/or FMN-binding domains as genomic markers, we previously computationally mined 31,910 genomes representative of the diversity of prokaryotic life and found that ~50% encoded machineries involved in flavinylation (16). We further observed that ~50% of genomes that encode ApbE flavinylation machineries lacked one of the previously characterized systems mentioned above. Consistent with this reflecting the existence of uncharacterized flavinylation-based electron transfer systems, experimental characterization of proteins encoded by genes that co-localize with *apbE* revealed that extracytosolic flavinylation occurs in proteins with a variety of different domain topologies and is associated with novel transmembrane proteins that link redox pools in the membrane to the extracytosolic space (16). Previous studies thus establish that ApbE flavinylation is a central component of prokaryotic extracytosolic redox activities but highlight limitations in our understanding of the molecular basis of its function across bacteria.

The recent development of AlphaFold, an artificial intelligence-powered protein structure prediction tool, has created new opportunities for high-throughput analysis of protein structures with great speed and accuracy (17, 18). The coupling of AlphaFold structural models with gene co-localization analyses has the potential to provide a powerful tool to discover and characterize novel protein functions. Here, we sought to couple these approaches to address limitations in our understanding of the mechanism and scope of flavinylation-based electron transfer. Our results demonstrate the AlphaFold models complement gene co-localization inference of protein function and reveal multiple facets of flavinylation-based electron transfer throughout prokaryotic life.

## RESULTS

### AlphaFold models reveal a diverse context of ApbE flavinylation in proteins

**Fig 1:**
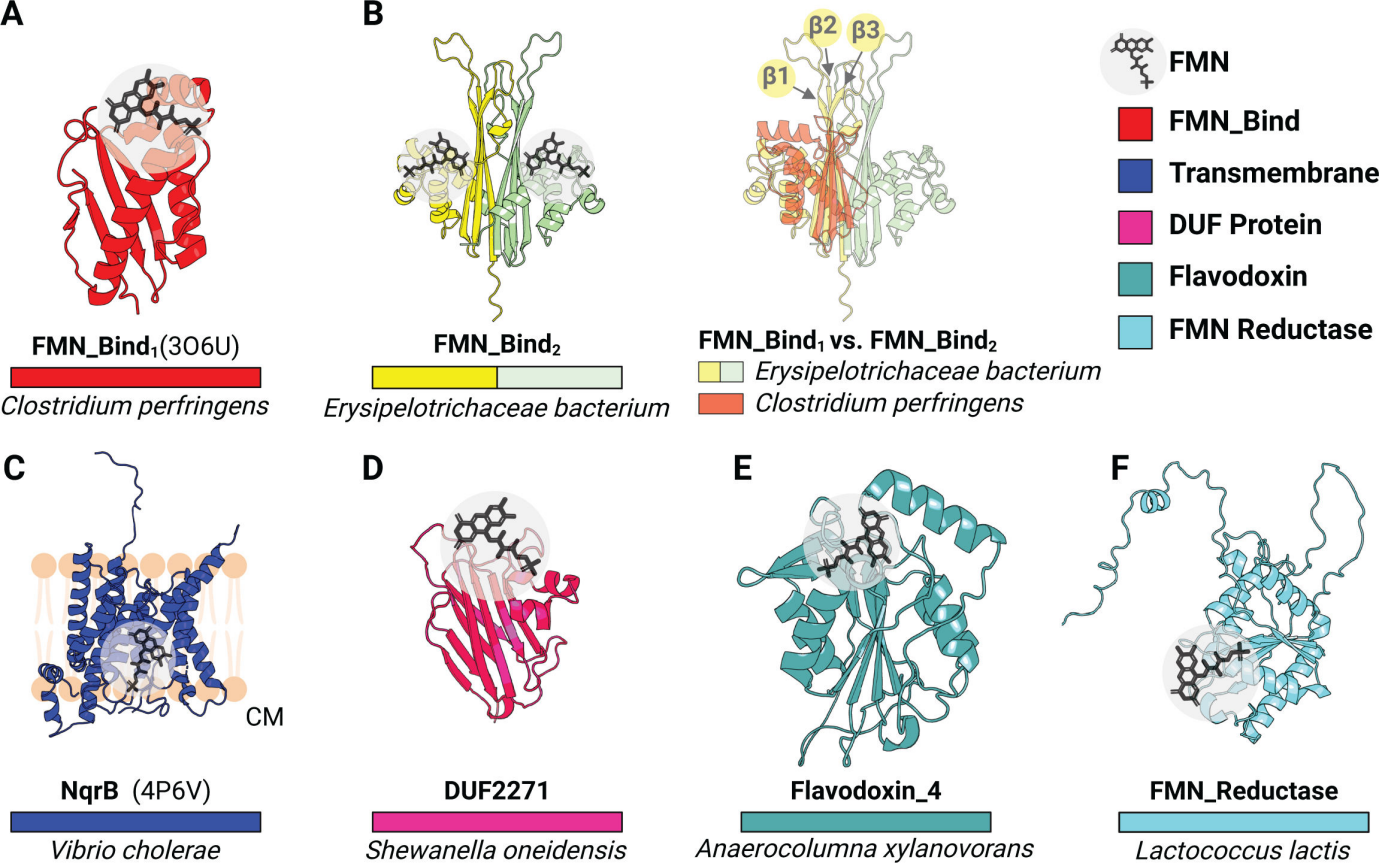
Structural context of ApbE flavinylation sites. (**A**) Previously resolved crystal structure of a flavinylated monomeric protein from *C. perfringens* (FMN-Bind_1_, PDB: 3O6U). (**B**) AlphaFold model of a double-flavinylated protein from *Erysipelotrichaceae bacterium* containing 2 FMN-bind_2_ domains (left) and its structural alignment with FMN-bind_1_ (right). Arrows highlight β1, β2, and β3 strands. (**C**) Previously resolved crystal structure of the B subunit from the Nqr complex (PDB: 4P6V). (**D**) AlphaFold model of a *Shewanella oneidensis* protein with a flavinylated DUF2271 domain. (**E and F**) AlphaFold models of flavinylated Flavodoxin_4 (**E**) and flavinylated FMN reductase (**F**). Gray circles highlight the position of the flavinylated amino acid.

To address general principles of electron transfer through ApbE-flavinylated proteins, we analyzed representative AlphaFold models of previously identified ApbE flavinylated domains (FMN-bind, NqrB/RnfD, and DUF2271) and compared these to experimentally characterized structures (16). The resulting collection of structures reveals a diverse context of flavinylation that varies across the different classes of flavinylated proteins. Flavinylated FMN-bind, NqrB/RnfD, and DUF2271 domains each possess a distinct fold and a unique structural context of the flavinylation site (Fig. 1). FMN-bind and DUF2271 are small soluble and generally non-descript domains with distinct folds (Fig. 1A and D). NqrBs/RnfDs are transmembrane proteins which typically serve as subunits within larger multi-protein complexes in the cytosolic membrane (Fig. 1C) (19). Analyses of structural models of confirmed flavinylation substrates thus provide evidence of a variable context of flavinylation sites that partially reflects distinctions in domain cellular localization between the cytosolic membrane, the periplasm, and the outer membrane.

**Fig 2:**
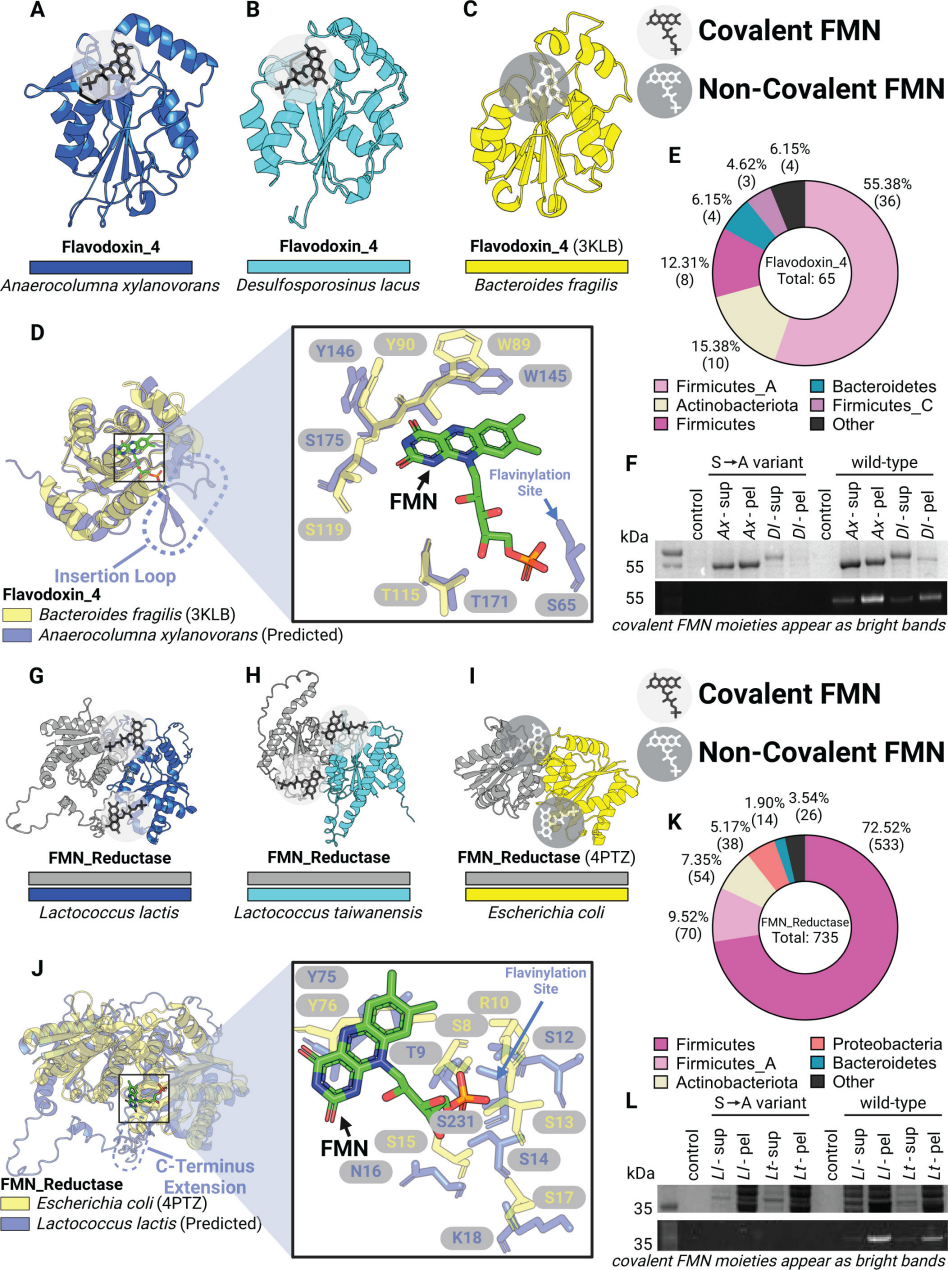
ApbE flavinylation evolved from non-covalent flavoproteins. (**A and B**) AlphaFold models for flavinylated Flavodoxin_4 proteins from *Anaerocolumna xylanovorans* (**A**) and *Desulfosporosinus lacus* (**B**). (**C**) Previously resolved crystal structure of a Flavodoxin_4 with non-covalently bound FMN (PDB: 3KLB). (**D**) Structural alignments of Flavodoxin_4 proteins with and without predicted flavinylation site. Dashed oval highlights an inserted loop that is lacking in 3KLB (left). Right panel shows zoom-in view of non-covalent FMN molecule from 3KLB and surrounding residues. Arrow indicates the serine residue in flavinylated Flavodoxin_4 responsible for covalent FMN binding. (**E**) Taxonomic distribution of Flavodoxin_4 proteins in bacteria. (**F**) SDS-PAGE gel image of purified flavinylated Flavodoxin_4 from *A. xylanovorans* and *D. lacus*, visualized under UV. (**G and H**) AlphaFold models for flavinylated FMN reductases from *Lactococcus lactis* and *Lactococcus taiwanensis*. (**I**) Previously resolved crystal structure of a FMN reductase with non-covalently bound FMN (PDB: 4PTZ). (**J**) Structural alignments of FMN reductase proteins with and without covalent FMN binding. Dashed oval highlights a C-terminus extension that is lacking in 4PTZ (left). Right panel shows zoom-in view of non-covalent FMN molecule from 4PTZ and surrounding residues. Arrow indicates the serine residue in flavinylated FMN reductase responsible for covalent FMN binding. (**K**) Taxonomic distribution of FMN reductases. (**L**) SDS-PAGE gel image of purified flavinylated Flavodoxin_4 proteins from *L. lactis* and *L. taiwanensis*, visualized under UV. Covalent FMN moieties appear as bright bands on SDS-PAGE visualized under UV.

We also observed that structural distinctions distinguish domains from different proteins within ApbE substrate classes. For example, the most common flavinylated domain, the FMN-bind domain, exhibits two distinct structural subtypes. The more common FMN-bind_1_ subtype comprises a compact ~120-amino acid structural core. The ~160-amino acid FMN-bind_2_ domain is less common and sometimes found in multiple copies within proteins. Proteins with multiple FMN-bind_2_ domains often contain an even number of domains (two or four) and an AlphaFold structure of the *Erysipelotrichaceae bacterium* two-FMN-bind_2_ domain protein provides an explanation for this pattern. Relative to the FMN-bind_1_ domain, FMN-bind_2_ domains have insertions between β1-/β2-strands and the β3-strand/α1-helix that extends the β-sheet face of the domain (Fig. 1B). This β-sheet face is predicted to interact with a homologous β-sheet on a neighboring FMN-bind_2_ domain within multi-FMN-bind_2_ domain proteins to produce a pseudo-symmetrical unit with two flavinylated sites (Fig. 1B). Distinctions in the FMN-bind_1_ and FMN-bind_2_ domain sequence thus seem to establish unique one- and two- flavinylated structural units, respectively.

### Structural models suggest an evolutionarily link between non-covalent flavoproteins and ApbE flavinylation

To further expand our analyses of ApbE flavinylation substrates, we next performed comparative genomic analyses to mine the Genome Taxonomy Database (GTDB) collection of 47,894 diverse bacterial and archaeal genomes and metagenome-assembled genomes (20). Through this analysis, we identified two groups of candidate flavinylation substrates that co-localize with *apbE* genes and contain an ApbE-like flavinylation motif sequence. Both candidates are related to characterized flavoproteins that contain a non-covalently bound flavin co-factor. The first candidate includes proteins within the Flavodoxin_4 protein family (Pfam accession PF12682) that have an insertion with flavinylation motif-like sequence internal to the Flavodoxin_4 domain (Fig. 1E and 2A). The second candidate includes proteins within the FMN_red protein family (Pfam accession PF03358) that have an extended C-terminal region that contains one to two flavinylation motif-like sequences (Fig. 1F and 2G). ApbE-associated proteins from both the Flavodoxin_4 and FMN_red protein families are predominately encoded by members of the Firmicutes phylum (Fig. 2E and K).

To assess the likelihood of identified flavinylation motif-like sequences representing bona fide flavinylation sites, we compared ApbE-associated Flavodoxin_4 and FMN_red AlphaFold models to crystal structures of homologous proteins bound to a non-covalent flavin co-factor. For both Flavodoxin_4 and FMN_red structures, we observed that the core flavin-binding domain is structurally similar irrespective of putative flavinylation status (Fig. 2). AlphaFold models of the ApbE-associated Flavodoxin_4 proteins from *Anaerocolumna xylanovorans* (Fig. 2A; National Center for Biotechnology Information [NCBI] accession SHO45324.1) and *Desulfosporosinus lacus* (Fig. 2B; NCBI accession WP_073032509.1) resemble a crystal structure of a homologous non-covalent flavin-binding protein (PDB: 3KLB) from *Bacteroides fragilis* (Fig. 2C). However, the ApbE-associated Flavodoxin_4 proteins include an insertion between the 1β-strand and the 1α-helix that contains the predicted flavinylated serine (Fig. 2D). Strikingly, the predicted flavinylated serine/threonine in *A. xylanovorans* Flavodoxin_4 is perfectly positioned for the covalently bound flavin to engage the conserved flavin-binding site (Fig. 2D).

A similar pattern is evident in the ApbE-associated FMN_red proteins. The crystal structure of an *Escherichia coli* FMN_red protein (PDB accession: 4PTZ) reveals a homodimer with symmetric flavin-binding sites at the dimerization interface (Fig. 2I). AlphaFold-multimer structures of the ApbE-associated FMN_red proteins from *Lactococcus lactis* (Fig. 2G; NCBI accession WP_021723379.1) and *Lactococcus taiwanensis* (Fig. 2H; NCBI accession WP_205872264.1) reveal a similar dimerization mode. Within the *L. lactis* model, predicted flavinylation sites are positioned on an unstructured C-terminal extension proximal to the conserved flavin-binding sites (Fig. 2J). AlphaFold models thus provide evidence of the structural congruity between and ApbE-associated FMN_red and Flavodoxin_4 flavinylation motif-like sequences and structurally conserved flavin-binding sites.

**Fig 3:**
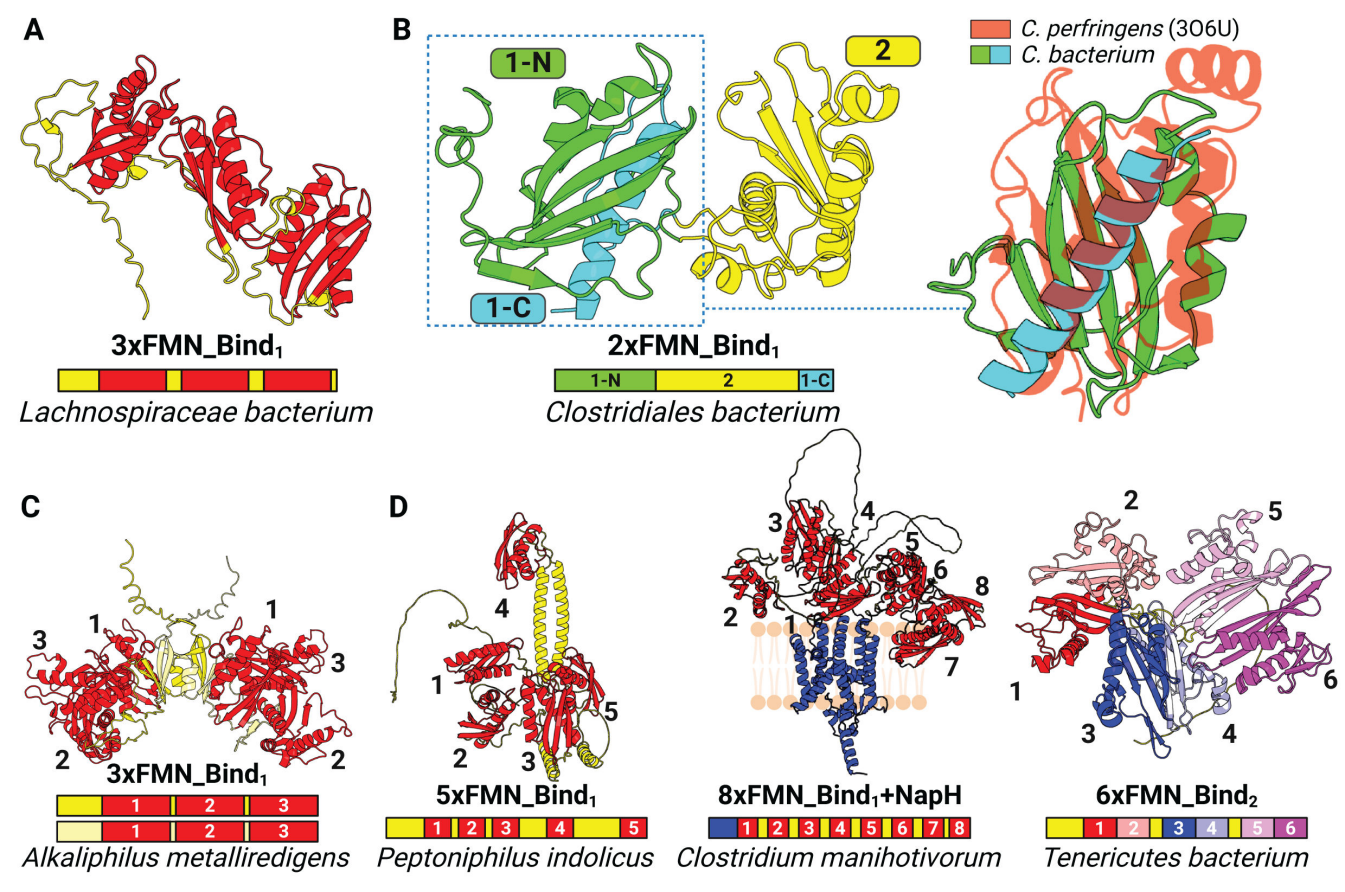
AlphaFold models of multi-flavinylated proteins. (**A**) Predicted structure of a “beads-on-a-string-like” 3×FMN-bind_1_ protein. (**B**) Predicted structure of a circularly permuted double-flavinylated protein. N-terminus and C-terminus regions predicted to form the first FMN-bind_1_ domain are green and cyan, respectively, whereas the second FMN-bind_1_ domain is highlighted in yellow (left). Circularly permuted FMN-bind_1_ domain is structurally aligned with previously resolved crystal structure of a stand-alone FMN-bind_1_ protein (PDB: 3O6U; right). (**C**) Predicted structure of a dimerized 3×FMN-bind_1_ protein. (**D**) Predicted structures of flavinylated proteins with more than three FMN-bind_1_ or FMN-bind_2_ domains.

As our analysis of AlphaFold structures suggested that flavinylation sites could secure flavins within established flavin-binding sites, we sought to address whether ApbE-associated FMN_red and Flavodoxin_4 domains were novel flavinylation substrates. We co-expressed *A. xylanovorans* and *D. lacus* Flavodoxin_4 proteins, as well as *L. lactis and L. taiwanensis* FMN_red proteins with their cognate *apbE* in *E. coli*. To address the specificity of flavinylation, we also expressed variants of these proteins with alanine point mutations at the predicted flavinylation site. SDS-PAGE analyses confirmed that FMN_red and Flavodoxin_4 proteins were flavinylated and that this required a serine/threonine at the predicted flavinylation site (Fig. 2F and L). These findings thus highlight the utility of AlphaFold models in guiding protein function predictions, expand the repertoire of ApbE substrates, and suggest that, at least in some instances, flavinylated proteins evolved through the acquisition of a flavinylation motif at a non-covalent flavin-binding site.

### Multi-flavinylated proteins exhibit unique features and marked structural heterogeneity

Having identified flavinylated structural motifis, we next sought to address how they assemble into higher-order structures. We previously identified a group of proteins predicted to have multiple (as many as 13) flavinylation sites (16). These functionally uncharacterized multi-flavinylated proteins have highly variable sequences/number of FMN-bind domains but are generally predicted to be extracyotosolic. Expression and flavinylation of multi-flavinylated proteins have previously been observed (21). The function of multi-flavinylated proteins is unclear but could be analogous to multiheme cytochromes, which assemble a path of redox-active co-factors that facilitate electron transfer over longer extracytosolic distances (22).

To generate insight into the structure and function of multi-flavinylated proteins, we analyzed AlphaFold models of proteins with >2 FMN-bind domains. We observed that multi-flavinylated AlphaFold models frequently possess predicted “beads-on-a-string-like” structures, with minimal predicted interactions predicted between FMN-bind_1_ domains (Fig. 3A; Fig. S1). Despite this general organization, striking structural features distinguish subsets of multi-flavinylated AlphaFold models. We identified one group of multi-FMN-bind_1_ proteins that are circularly permuted, with the displacement of the C-terminal helix of the N-terminal FMN-bind_1_ domain to the C-terminus of the protein creating a multi-domain structure that is predicted to interact with the N-terminal domain (Fig. 3B). Another group of three-domain FMN-bind_1_ proteins is predicted to have pseudo threefold symmetry that generates a structure resembling a three-petaled flower. These proteins further contain an N-terminal dimerization domain (Pfam accession PF07833) that produces a homodimer with a predicted two-flower bouquet-like structure (Fig. 3C). A final example of multi-flavinylated AlphaFold structures is illustrated by a group of proteins defined by a coiled-coil core decorated with multiple FMN-bind_1_ domains in a haphazard-appearing manner (Fig. 3D). These observations thus reveal a structural heterogeneity that may suggest a diversity of functions of multi-flavinylated proteins.

### Flavinylation is associated with diverse transmembrane electron transfer mechanisms

**Fig 4:**
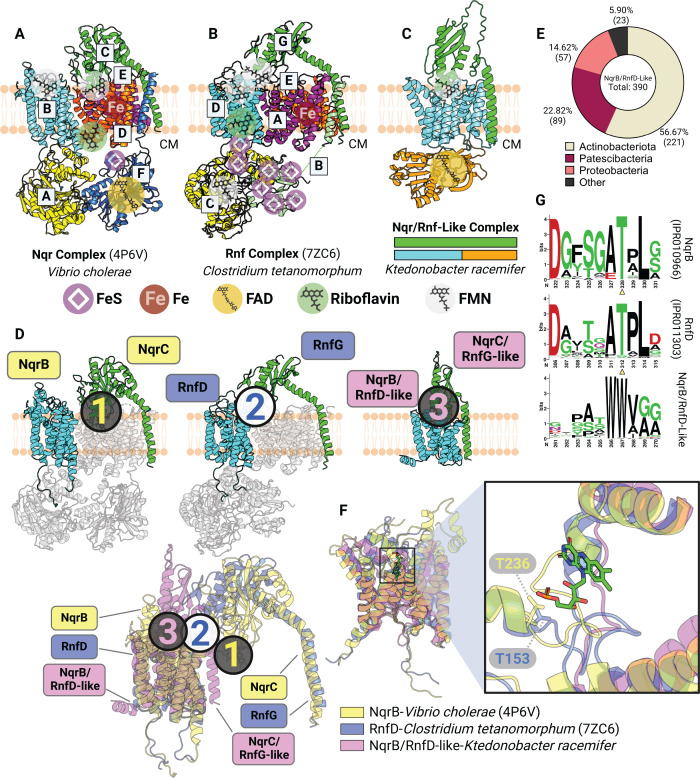
Flavinylation-associated transmembrane proteins exhibit structural heterogeneity. (**A and B**) Previously resolved crystal structures of the Nqr complex (A, PDB: 4P6V) and the Rnf complex (B, PDB: 7ZC6). The cytoplasm (CM) is indicated. (**C**) AlphaFold-multimer model of the Nqr/Rnf-like complex containing a transmembrane subunit and a membrane-anchored extracytosolic subunit that share homology with corresponding subunits in Nqr and Rnf systems. (**D**) Structural alignment of Nqr, Rnf, and Nqr/Rnf-like complexes based on transmembrane subunits homologous across the three complexes (cyan). Subunits that do not share homology to subunits of the Nqr/Rnf-like complex are transparent (top). Predicted or confirmed FMN moieties are highlighted by circled numbers, color-coded by their corresponding complex (bottom). (**E**) Taxonomic distribution of the NqrB/RnfD-like complexes in bacteria. (**F**) Structural alignment of NqrB, RnfD, and NqrB/RnfD-like subunits (left) with zoom-in view showing the FMN moiety from NqrB and the Thr residue in NqrB or RnfD responsible for FMN-binding (right). (**G**) HMM logos showing conserved Thr residues in NqrB and RnfD but not in the NqrB/RnfD-like subunit.

Having addressed the structural context of flavinylation across bacteria, we next sought to clarify the basis of transmembrane electron transfer required for the function of flavinylated proteins. We previously identified five characterized and five uncharacterized electron transfer systems that co-localize on bacterial genomes with flavinylated proteins and which presumably utilize distinct mechanisms to mediate electron transfer from cytosolic or membrane donors to extracytosolic flavinylated domains (16). Structures of the Nqr complex, the Rnf complex, and the *Pseudomonas aeruginosa* PepSY-like protein FoxB have been experimentally characterized, but the structural basis of other flavinylated-associated membrane electron transfer domains remains unknown (23–27). To clarify the context of transmembrane electron transfer, we generated AlphaFold or AlphaFold-multimer models for representatives of the remaining systems (Fig. 4A through C and 5A through C).

**Fig 5:**
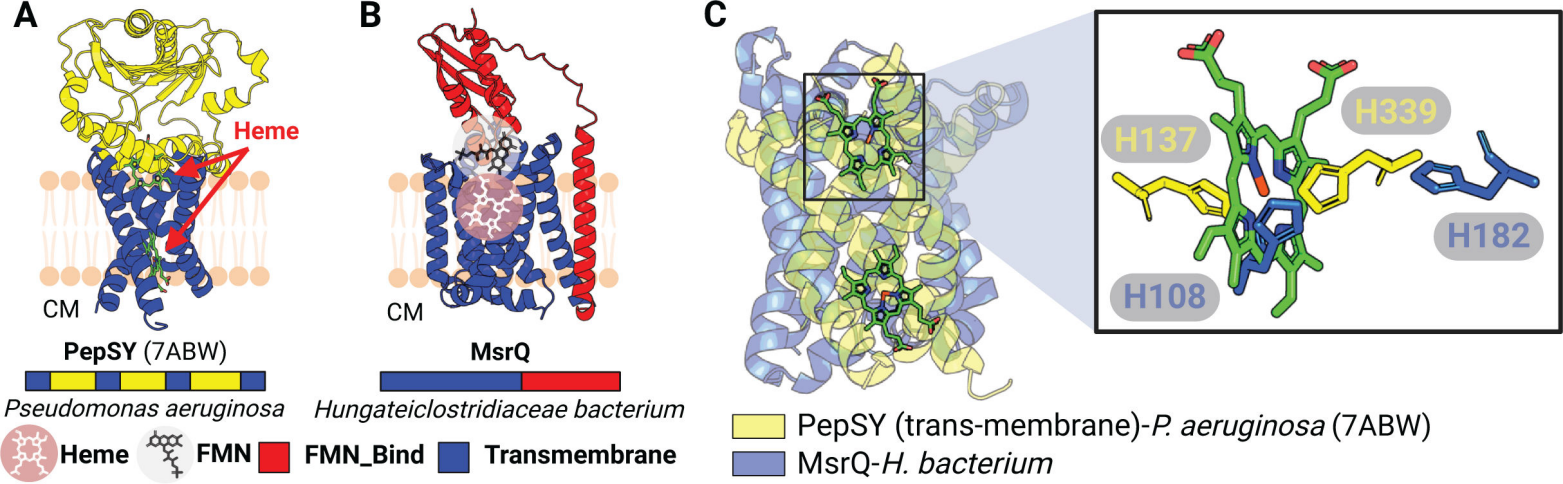
Membrane cytochromes associated with flavinylated proteins. (**A**) Previously resolved crystal structure of the PepSY complex (PDB: 7ABW). The cytoplasm (CM) is indicated. (**B**) AlphaFold model of a protein containing the transmembrane cytochrome MsrQ (red) and a FMN-Bind_1_ domain (red). (**C**) Structural alignment between transmembrane segments of PepSY and MsrQ (left) with zoom-in view on the heme and axial histidines from PepSY and MsrQ.

### Nqr, Rnf, and Nqr/Rnf-like complexes exhibit distinct but related paths for electron transfer

We next sought to understand the basis of electron transfer in the group of related multi-subunit complexes with flavinylated subunits. Previously reported structures of the six-subunit Nqr complex reveal a semicircular electron transfer pathway in which electrons travel from the cytosolic NADH to the extracytosolic NqrC flavinylation site, to the quinone terminal electron acceptor on the cytosolic side of the membrane (Fig. 4A) (23, 24). This reaction is coupled to the transfer of ions across the membrane and the creation of an electromotive force. Rnf is evolutionarily related to Nqr and possesses four homologous subunits but distinct substrate- and product-binding subunits that enable electron transfer between ferredoxin and NAD^+^. Recently reported cryoelectron microscopy structures provide evidence that RNF possesses a similar structure and mechanism as Nqr (Fig. 4B) (26, 27).

Our previous study identified Nqr/Rnf-like complexes as a distinct group of flavinylation-associated transmembrane subunits related to Nqr and Rnf (Fig. 4C and E) (16). These gene clusters contain only two apparent subunits, with one extracytosolic subunit sharing homology with NqrC and RnfG. The second subunit has an N-terminal membrane domain homologous to NqrB and RnfD and a cytosolic C-terminal NAD-binding domain (Pfam accession PF00175). In contrast to the semicircular Nqr and Rnf electron transfer path described above, we previously proposed that Nqr/Rnf-like complexes unidirectionally transfer electrons from NAD(P)H to extracytosolic electron acceptors (16). A high-confidence AlphaFold-multimer model *Ktedonobacter racemifer* Nqr/Rnf-like complex predicts that the two subunits of the Nqr/Rnf-like complex intimately interact with each other, while similar interactions between their corresponding homologs in the Nqr or Rnf complexes are absent (Fig. 4A through D). In addition, the transmembrane domain in the Nqr/Rnf-like complex lacks a flavinylation site that is conserved in NqrB and RnfD (Fig. 4F and G). Strikingly, the unique interaction between the two subunits in the Nqr/Rnf-like AlphaFold model brings the extracytosolic subunit’s flavinylation site in close proximity to the apparent non-covalent flavin-binding site in the transmembrane subunit. This observation suggests that flavin covalently bound to the extracytosolic subunit may conformationally sample the non-covalent flavin-binding site in the transmembrane domain (Fig. 4D). Collectively, these observations suggest a dynamic evolutionary history resulted in marked functional distinctions between related Nqr, Rnf, and Nqr/Rnf-like systems.

### Flavinylation-associated transmembrane cytochromes exhibit structural conservation

We next sought to address the basis of flavinylation-associated transmembrane cytochromes. PepSY-like and MsrQ-like transmembrane proteins are predicted to contain heme co-factors that transfer electrons across membranes. A recently reported crystal structure of the *Pseudomonas aeruginosa* PepSY-like protein revealed that it has two heme-binding sites and that each site contains two highly conserved histidines that coordinate heme binding (25) (Fig. 5A). Despite low sequence identity, MsrQ-like AlphaFold structures exhibit considerable structural homology to the *Pseudomonas aeruginosa* PepSY-like protein, including two highly conserved histidines that come together to form a similar predicted heme-binding site (Fig. 5B and C). These structures thus demonstrate a similar transmembrane core that is conserved within the flavinylation-associated cytochrome electron transfer apparatuses.

### DUF4405-like proteins encompass a widespread class of flavinylation- and ferrosome-associated cytochromes

**Fig 6:**
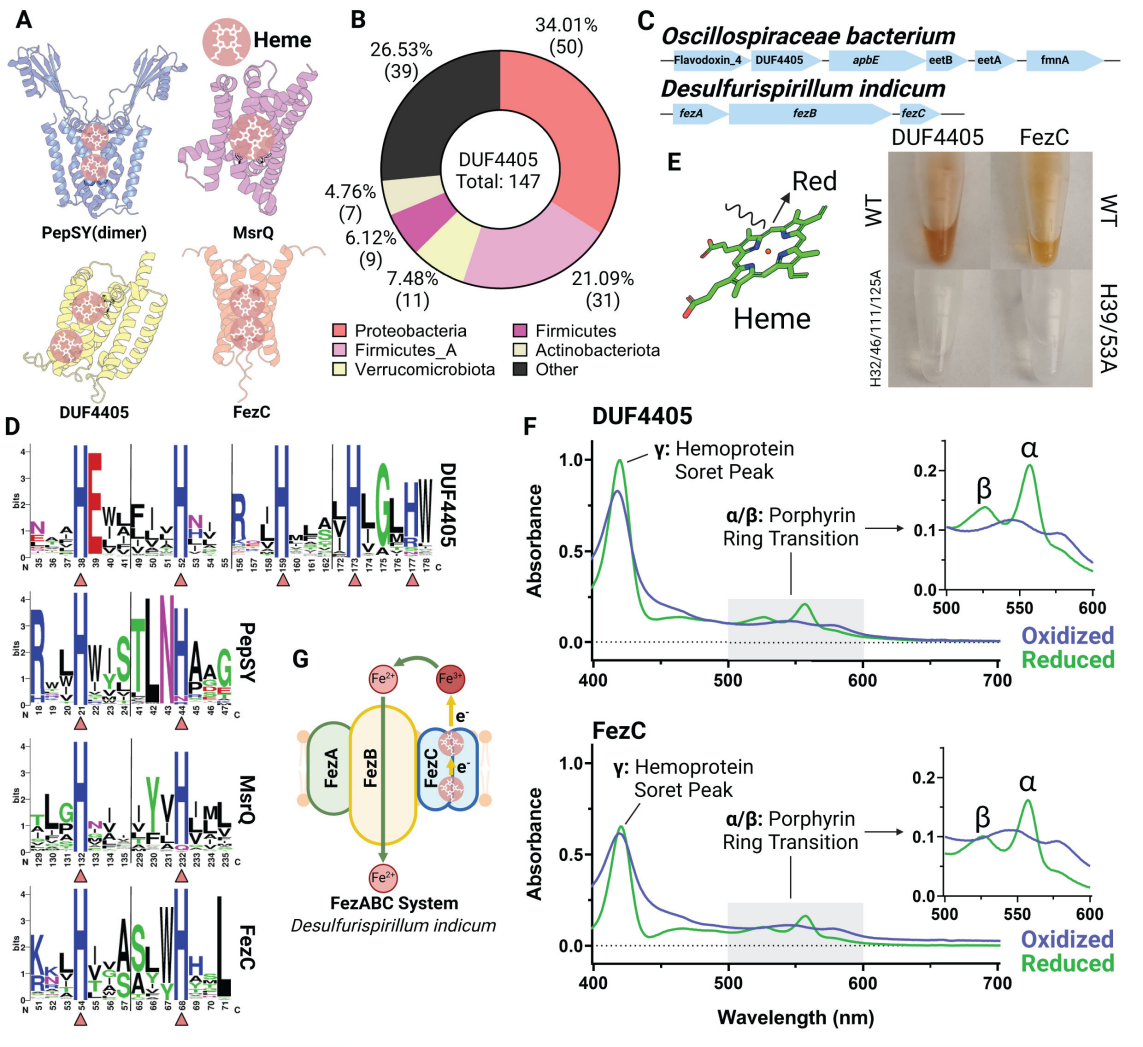
DUF4405 and FezC are novel cytochromes. (**A**) AlphaFold models of MsrQ (homodimer), DUF4405, and FezC (homodimer). Histidine residues (His) responsible for heme binding are highlighted. (**B**) Taxonomic distribution of DUF4405 genes contained in a cluster with *apbE*. (**C**) Gene clusters in a *Oscillospiraceae bacterium* or *Desulfurispirillum indicum* encoding DUF4405 or FezC, respectively. (**D**) HMM logos showing conserved heme-binding His residues in DUF4405, PepSY, MsrQ, and FezC. Triangles highlight His residues shown in panel **A**. (**E**) Purified DUF4405 and FezC proteins. (**F**) UV spectrums of DUF4405 (top) and FezC (bottom) showing absorption peaks characteristic of heme B binding. (**G**) Possible role of FezC in iron transport within ferrosomes.

Having defined the structural features responsible for flavinylation-associated membrane electron transfer, we next asked whether these insights could be leveraged to enable the discovery of novel proteins with analogous functionalities. We reasoned that such proteins would likely localize to genes clusters that contain *apbE* but lack a characterized flavinylation-associated electron transfer mechanism. We performed comparative genomic analyses mining *apbE* gene clusters that lack a known electron transfer mechanism and identified a group of transmembrane proteins with a DUF4405 domain of unknown function that frequently co-localized with *apbE* in Proteobacteria and Firmicutes species (Fig. 6A and B). Consistent with these DUF4405s functioning in flavinylation-based electron transfer, we observed gene clusters containing *apbE* and DUF4405 genes also often encoded genes for a flavinylated Flavodoxin_4 domain and a transporter that provisions *Listeria monocytogenes* with extracytosolic flavins (Fig. 6C) (28).

To assess whether identified DUF4405 proteins might be involved in transmembrane electron transfer, we analyzed AlphaFold structures of representative proteins (Fig. 6A). These structures revealed that DUF4405 possesses a six-transmembrane structure with four centrally located histidines arranged strikingly similarly to PepSY-like and MsrQ-like structures (Fig. 6D). Despite a low overall sequence homology to PepSY-like or MsrQ-like proteins, an inspection of sequence conservation within the DUF4405 domain revealed that its centrally located histidines are similarly the most highly conserved amino acids (Fig. 6D). These analyses thus establish that DUF4405 resembles flavinylation-associated cytochromes.

To test the hypothesis that DUF4405 represents a novel class of cytochromes, we recombinantly expressed the *Oscillospiraceae bacterium* DUF4405 protein. Strikingly, DUF4405 overexpression conferred *E. coli* cells with a pinkish hue typical of heme-binding protein (Fig. 6E). Purified DUF4405 retained this color, and spectroscopic analyses revealed absorbance peaks consistent with heme B binding (Fig. 6F). These results thus demonstrate that DUF4405s represent a novel class of cytochromes frequently associated with flavinylation-associated electron transfer.

### FezC is a ferrosome cytochrome

As only a minority of proteins with DUF4405 domains co-localize with *apbE*, we wondered whether the identification of DUF4405 as a cytochrome might clarify the function of other DUF4405 proteins. A previous reference to DUF4405 noted that the *Desulfovibrio magneticus* ferrosome protein FezC possesses sequence homology to DUF4405 proteins (29). Ferrosomes are recently discovered membrane-enclosed organelles that act as an intracellular store of iron within some bacteria (29, 30). As flavinylated systems commonly facilitate iron transport across membranes and this activity could be directly relevant for ferrosomes (which contain a putative ferrous iron transporter, FezB), we reasoned that the functionally uncharacterized FezC might be a DUF4405-like cytochrome. Indeed, an AlphaFold model revealed that FezC could form DUF4405-like heme-binding sites via homodimerization, and recombinant FezC exhibited cytochrome-like properties similar to DUF4405 (Fig. 6E and F). These results establish that FezC is a cytochrome and suggest that it may play a role in modulating iron redox status to facilitate iron transport into and/or out of ferrosomes (Fig. 6G).

## DISCUSSION

The importance of AbpE flavinylation for prokaryotic extracytosolic redox activities has become increasingly apparent in recent years. In this study, we combine comparative genomic context analysis of flavinylation-associated gene clusters with AlphaFold structural modeling to explore the molecular basis of flavinylation-associated electron transfer. Our findings showcase how recent advances in protein structural modeling enabled by AlphaFold can be leveraged for discovery and provide evidence that ApbE flavinylation is involved in a wide range of cellular processes.

By examining structural models of proteins encoded in flavinylation-associated gene clusters lacking a predicted transmembrane electron transfer apparatus, we identify DUF4405 as a putative electron-transferring cytochrome. Broadening these analyses, we find that related cytochromes include ferrosome components with obvious potential roles in modulating the redox state in these iron-containing organelles. These findings highlight how the iterative application of comparative genomic analyses and structural modeling can enable unpredictable protein functional attributions.

Our structural analysis of proteins encoded on flavinylation-associated gene clusters led to the discovery of two classes of ApbE flavinylated proteins (flavodoxin and FMN_red) that are closely related to unflavinylated flavoproteins (i.e., which non-covalently bind their flavin co-factor). These findings have implications for our understanding of the evolution and significance of protein flavinylation, demonstrating that, at least in some cases, flavinylation may have emerged as an evolutionary addition to unflavinylated precursor proteins. Moreover, the observation that extracytosolic flavodoxin proteins exhibit signs of flavinylation (in contrast to unflavinylated cytosolic members of the family) is consistent with the main role of ApbE flavinylation being to prevent flavin diffusion and loss in extracytosolic space.

In summary, our study demonstrates how comparative genomic analyses coupled with AlphaFold protein structure analyses can be leveraged to infer novel protein functions. Our results provide new insight into the structural context of ApbE flavinylation and suggest that this modification may play a broad role in bacterial biology. Future experiments will be needed to fully understand the function of ApbE flavinylation and its role in bacterial physiology.

## MATERIALS AND METHODS

### Identification of flavinylated protein candidates

The flavinylation-associated proteins FMN_red, DUF4405, and flavodoxin were identified by searching their Pfam accession numbers (PF03358, PF14358, and PF12682, respectively) in the proteomes from 47,894 functionally annotated bacterial and archaeal genomes from the GTDB (release 202) (20). FMN-bind_2_ domains were identified through BLAST searches. Briefly, protein sequences were functionally annotated based on the Pfam accession number (Pfam database v.33.0) (31) of their best match using Hmmsearch (v.3.3.2, *E* value cutoff of 0.001) (32). The five genes downstream and upstream of genes FMN_red, DUF4405, or flavodoxin were collected for further analyses. InterPro accession numbers, taxonomic assignments, and amino acid sequences of flavinylated candidates presented in this study are included in Table S1.

### Protein model prediction by AlphaFold2

Predicted three-dimensional models for selected flavinylated protein monomers or complexes were generated using AlphaFold2 and AlphaFold2-multimer (ColabFold v.1.5.2) (17, 18, 33). Model metrics are provided in Fig. S1. PDB files containing predicted structures were visualized, examined, or aligned using PyMOL Molecular Graphics System, Version 3.0 (Schrödinger, LLC).

### *In vitro* confirmation of flavodoxin and FMN reductase flavinylation

#### *E. coli* expression strains

DNA fragments containing wild-type or point mutant ORFs of flavodoxins (*Anaerocolumna xylanovorans*, NCBI accession number SHO45324.1; *Desulfosporosinus lacus*, NCBI accession WP_073032509.1) and FMN reductases (*Lactococcus lactis*, NCBI accession WP_021723379.1; *Lactococcus taiwanensis*, NCBI accession WP_205872264.1) were synthesized using IDT gBlocks (Integrated DNA Technologies). Primers with overhanging sequences homologous to either the 5′ or 3′ end of target gene fragments were used to linearize pMCSG53 expression vectors at the multiple cloning sites through PCR reactions (Q5 High-Fidelity 2X Master Mix, New England Biolabs). Amplicons were subsequently gel-extracted (Wizard SV Gel and PCR Clean-Up System, Promega), quantified, and combined with corresponding gene inserts in Gibson reactions (NEBuilder HiFi DNA Assembly Master Mix, New England Biolabs) to allow integration of targeted genes. Expression constructs were then transformed into *E. coli* BL21, and successful transformants were selected on lysogeny broth (LB) agar containing 100 mg/mL of carbenicillin. LB cultures of transformant colonies were supplemented with 15% (wt/vol) glycerol and stored in −80°C until use.

#### Purification of FMN transferase ApbE from *Listeria monocytogenes*

To ensure consistent flavinylation activity, we developed an *in vitro* flavinylation assay using the previously characterized FMN transferase ApbE protein encoded by *Listeria monocytogenes* 10403S (lm_ApbE) (9). *E. coli* BL21 expression strains containing pMCSG53::*lm_apbe* expression constructs were generated through steps similar to those mentioned above with 6xHis-tag at the N-terminus. To purify lm_ApbE, overnight cultures of the expression strain were diluted to an optical density at 600 nm (OD_600_) of 0.05 in 1 L of LB and incubated at 37°C with aeration. After 2 h of incubation, a final concentration of 1 mM of isopropyl-β-D-thiogalactopyranoside (IPTG) was added to allow induction of lm_ApbE expression at 30°C overnight. Cell pellet was then collected through centrifugation at 7,000 × *g* for 15 min and frozen at −80°C overnight. The cell pellet was resuspended in a solution containing 50 mM of Tris-HCl, pH = 7.5, 300 mM of NaCl, and 10 mM of imidazole at a volume that is five times the weight of the cell pellet. The resulting mixture was lysed through sonication (8 × 30 s pulses) and cleared by centrifugation at 40,000 × *g* for 30 min. Supernatants of cell lysates were passed through a nickel bead column (Profinity IMAC Ni-Charged Resin, Bio-Rad) to allow binding of lm_ApbE-6×His, which was then eluted with 500 mM imidazole. Successful elution of lm_ApbE-6×His was confirmed through 12% SDS-PAGE. Filtrate samples were then purified using a ÄKTA pure chromatography FPLC system (Cytiva). Elution fractions containing lm_ApbE-6×His were subsequently concentrated (4,000 × *g*; Pierce Protein Concentrators PES, 10K MWCO, Thermo Scientific) and quantified using a spectrophotometer (DS-11 FX+ spectrophotometer, DeNovix).

#### *In vitro* expression and flavinylation of flavodoxin and FMN reductase candidates

To confirm *in vitro* covalent binding of FMN on target candidate proteins, overnight cultures of *E. coli* BL21 strains containing he corresponding expression vectors mentioned above were reinoculated in 3 mL of LB and grown in the presence of 1 mM IPTG with aeration at 30°C overnight. Overnight cultures were then diluted to an optical density of OD_600_ = 0.5 and centrifuged for 1 min at 21,100 × *g*. Resulting cell pellets were resuspended in 100 μL of lysis buffer (500 μg/mL of lysozyme, 300 mM of NaCl, and 10 mM of imidazole in 50 mM of Tris-HCl, pH = 7.5) and incubated on ice for 30 min. Cell lysates were then combined with 0.3 μM of lm_ApbE, 1 mM of FAD, and 5 mM of MgSO_4_ and incubated at 4°C overnight with rotation to enable flavinylation. Reaction mixtures were then separated into aqueous or solid phases by centrifugation at 21,100 × *g* for 1 min and subsequently incubated at 98°C for 10 min. Both aqueous and solid portions (resuspended in 100 μL of lysis buffer) were then run on 12% SDS-PAGE. To confirm successful flavinylation, we leveraged the UV resonance property of the isoalloxazine ring of the FMN moiety, which led to a bright band at the expected molecular weight for targeted proteins when the SDS-PAGE gel is visualized under UV (iBright 1500 imaging system, Invitrogen).

### *In vitro* confirmation of heme-binding activity in FezC and DUF4405

Cloning, expression, and purification of FezC (*Desulfurispirillum indicum*, WP_013506634.1) or DUF4405 (*Oscillospiraceae bacterium*, MBD5117352.1) were done in similar procedures as lm_ApbE, except that the solid phase of cell lysates was used for downstream purification because FezC and DUF4405 are membrane proteins. Proteins in the solid phase of cell lysates were solubilized using a previously published protocol (34). Briefly, pelleted cell lysates were resuspended in a solution containing 50 mM of Tris-HCl, pH = 7.5, 300 mM of NaCl, 10 mM of imidazole, and 1% (wt/vol) lauryldimethylamine oxide (LDAO) and were subsequently purified as previously described using nickel bead column and FPLC (eluted with 500 mM imidazole + 0.1% [wt/vol] LDAO). Heme-binding activity of purified FezC or DUF4405 was confirmed using a previously published protocol for pyridine hemochromagen assay (35). Briefly, samples containing 1 mg/mL of purified FezC or DUF4405 were mixed with a solution containing 0.2 M NaOH, 40% (vol/vol) pyridine, and 500-µM potassium ferricyanide to oxidize protein samples. Oxidized proteins were then measured for their absorbance at 300–700 nm. Samples were then combined with a reducing solution containing 0.5 M sodium dithionite in 0.5 M NaOH to acquire reduced FezC or DUF4405, which were then similarly examined for its absorbance at the same range of wavelength.

## SUPPLEMENTAL MATERIAL

Figure S1 msystems.00375-24-s0001.pdf Quality of AlphaFold-predicted structures.

Table S1 msystems.00375-24-s0002.xlsx Proteins referenced in this study.

ASM does not own the copyrights to Supplemental Material that may be linked to, or accessed through, an article. The authors have granted ASM a non-exclusive, world-wide license to publish the Supplemental Material files. Please contact the corresponding author directly for reuse.
