# Supplementary material for: Versatile roles of protein flavinylation in bacterial extracyotosolic electron transfer
Source: mSystems. 2024 Jul 23;9(8):e00375-24. doi: 10.1128/msystems.00375-24 (PMC11334425; doi:10.1128/msystems.00375-24)
Supplement: Figure S1 — Quality of AlphaFold-predicted structures. [file msystems.00375-24-s0001.pdf]

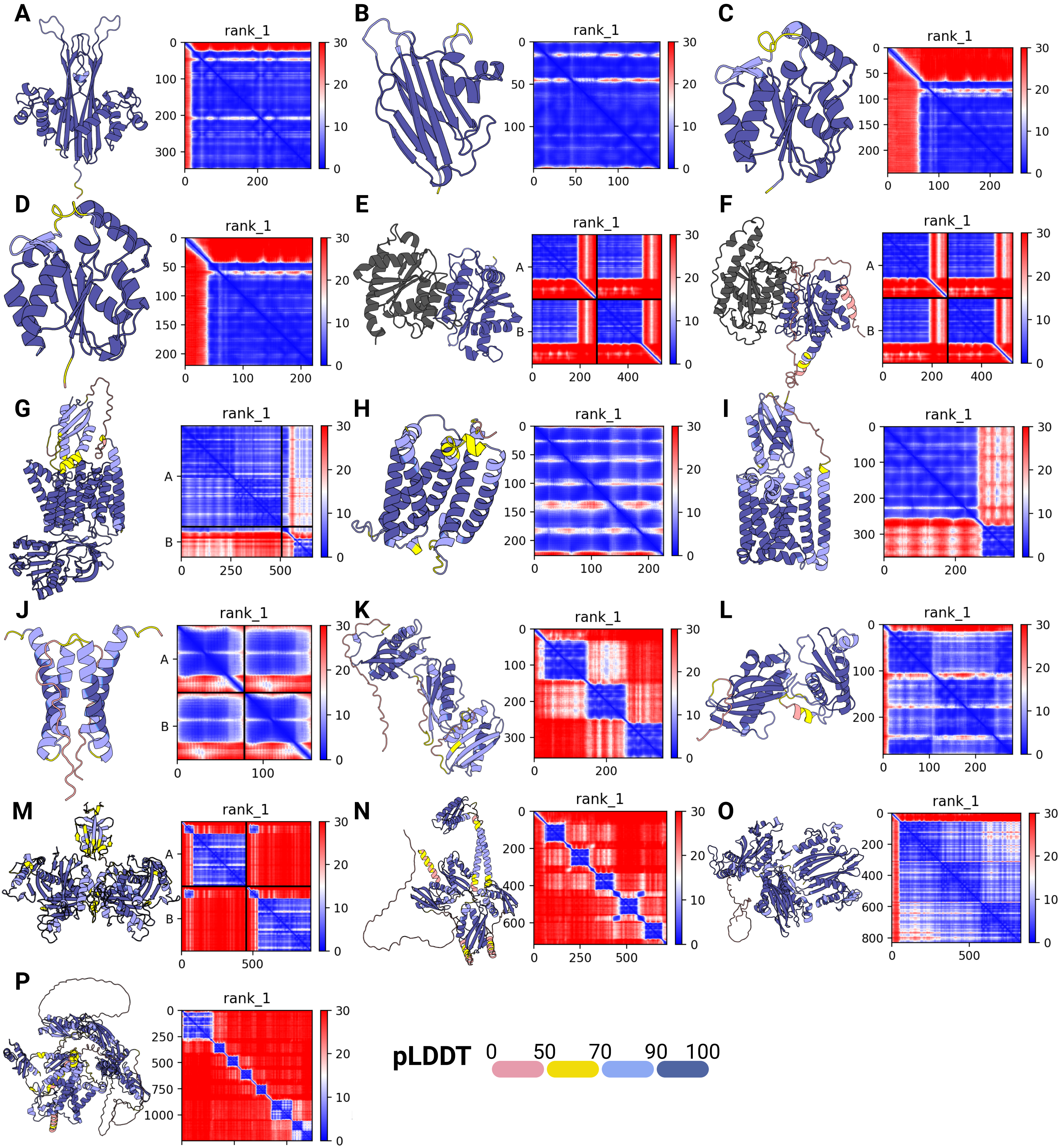

Supplementary Figure 1: Quality of AlphaFold-predicted structures.
(A-O) AlphaFold-predicted structures colored in predicted local distance difference test scores (pLDDT; left) as well as heatmaps of predicted aligned error (PAE; right). (A) FMN_Bind$_2$; (B) DUF2271; (C) Flavodoxin_4; (D) FMN_Reductase; (E) Flavodoxin_4 DL; (F) FMN_Reductase LT; (G) Nqr/Rnf-Like; (H) DUF4405; (I) MsrQ; (J) FezC; (K) 3xFMN_Bind$_1$; (L) 2xFMN_Bind$_1$; (M) 3xFMN_Bind$_1$; (N) 5xFMN_Bind$_1$; (O) 8xFMN_Bind$_1$; (P) 6xFMN_Bind$_2$.
